# CHPF Regulates the Aggressive Phenotypes of Hepatocellular Carcinoma Cells via the Modulation of the Decorin and TGF-β Pathways

**DOI:** 10.3390/cancers13061261

**Published:** 2021-03-12

**Authors:** Chiung-Hui Liu, Bo-Rui Wu, Ying-Jui Ho, Yin-Hung Chu, Wei-Cheng Hsu, To-Jung Tseng, Ju-Pi Li, Wen-Chieh Liao

**Affiliations:** 1 Department of Anatomy, Faculty of Medicine, Chung Shan Medical University, Taichung 40201, Taiwan; chiung@csmu.edu.tw (C.-H.L.); s0701009@gm.csmu.edu.tw (B.-R.W.); yinhung@csmu.edu.tw (Y.-H.C.); s0801044@gm.csmu.edu.tw (W.-C.H.); tjtseng@csmu.edu.tw (T.-J.T.); 2 Department of Medical Education, Chung Shan Medical University Hospital, Taichung 40201, Taiwan; 3 Department of Psychology, Chung Shan Medical University, Taichung 40201, Taiwan; yjho@csmu.edu.tw; 4 Department of Pathology, Faculty of Medicine, Chung Shan Medical University, Taichung 40201, Taiwan; 5 Department of Pediatrics, Chung Shan Medical University Hospital, Taichung 40201, Taiwan

**Keywords:** hepatocellular carcinoma, tumor microenvironment, chondroitin polymerizing factor, chondroitin sulfate, decorin

## Abstract

**Simple Summary:**

Altered extracellular chondroitin sulfate (CS) contributes to tumor progression in many cancers. CHPF is a key enzyme supporting the elongation of CS. Here we showed that CHPF was frequently downregulated in hepatocellular carcinoma (HCC) tumors compared with adjacent non-tumor tissues, and its downregulation was associated with poor overall survival. CHPF regulated aggressive phenotypes of HCC cells in vitro and in vivo, and the TGF-β pathway involved in the phenotypical changes. Mechanistically, CHPF modified CS on decorin (DCN), which could facilitate DCN accumulation surrounding HCC cells, and modulate activation of TGF-β pathway. Indeed, the expression of DCN were positively associated with CHPF levels in primary HCC tissue. The research proposed novel insights into the significance of CHPF, which modified DCN and modulated TGF-β signaling.

**Abstract:**

Aberrant composition of glycans in the tumor microenvironment (TME) and abnormal expression of extracellular matrix proteins are hallmarks of hepatocellular carcinoma (HCC); however, the mechanisms responsible for establishing the TME remain unclear. We demonstrate that the chondroitin polymerizing factor (CHPF), an enzyme that mediates the elongation of chondroitin sulfate (CS), is a critical elicitor of the malignant characteristics of HCC as it modifies the potent tumor suppressor, decorin (DCN). CHPF expression is frequently downregulated in HCC tumors, which is associated with the poor overall survival of HCC patients. We observed that restoring CHPF expression suppressed HCC cell growth, migration, and invasion in vitro and in vivo. Mechanistic investigations revealed that TGF-β signaling is associated with CHPF-induced phenotype changes. We found that DCN, as a TGF-β regulator, is modified by CHPF, and that it affects the distribution of DCN on the surface of HCC cells. Importantly, our results confirm that CHPF and DCN expression levels are positively correlated in primary HCC tissues. Taken together, our results suggest that CHPF dysregulation contributes to the malignancy of HCC cells, and our study provides novel insights into the significance of CS, which affects DCN expression in the TME.

## 1. Introduction

Hepatocellular carcinoma (HCC) occurs in 90.73% of men and 85.42% of women suffering from liver cancer; it is the third-leading cause of cancer-related deaths worldwide [1]. Surgical resection, transarterial chemoembolization, radiofrequency ablation, and liver transplantation are the common HCC treatment options; however, these approaches are only applicable in 30% of patients, and up to 50% of patients show relapse [2]. Invasiveness and metastasis of cancer cells are the predominant factors causing high HCC relapse rates. Accumulating evidence suggests that changes in stromal cells, immune cells, extracellular proteins, and glycosylation status establish a tumor microenvironment (TME) that facilitates HCC progression [3,4,5,6,7]. The complex interactions between cancer cells and the TME are difficult to directly interpret from genomic information of tumor cells; therefore, exploring the mechanisms underlying the activity of cancer cells and TME interactions in HCCs may provide important information for developing novel clinical diagnosis methods and treatment options.

Glycosaminoglycans (GAGs), which are unbranched polysaccharide chains in the extracellular matrix and on the cell surface, participate in various biological interactions with the TME. GAGs that covalently link to core proteins are known as proteoglycans (PGs), and certain types of GAGs such as hyaluronan occur as free chains. Recent studies have suggested that aberrant GAGs frequently accumulate in the TME of HCCs, and that may be correlated with disease progression. Certain specific modifications of GAGs are used as biomarkers for disease diagnosis and as pharmacological targets [8,9,10]. Chondroitin sulfate (CS) is a major type of GAG. In the past, the functions of CS chains were only considered to be associated with structural stabilization. Various growth factors, proteases, cytokines, chemokines, and adhesion molecules have been found to interact with CS chains; therefore, the importance of CS chains in disease progression has been reevaluated [11,12,13,14,15].

The biosynthesis of CS chains begins with the formation of a link between N-acetylgalactosamine (GalNAc) and a common tetrasaccharide structure at a serine residue on the core protein. The next polymerization (elongation) step is catalyzed by a group of enzyme complexes (composed by CHSY1, CHPF, CHPF2, and CHSY3), which have β1–3 glucuronosyltransferase and β1–4 N-acetylgalactosaminyltransferase activities [16,17,18,19]. A single CS chain can consist of up to 50 repeating glucuronic acid (GlcA)-GalNAc subunits, which are modified with sulfate groups at various positions. Furthermore, C5 epimerase (DSE and DSEL1) converts GlcA to iduronic acid within CS chains in certain cell types. Depending on the spectrotemporal expression of the polymerization and modification enzymes, CS chains can vary in lengths and numbers of sulfated units.

Several recent studies have revealed that these CS synthases and CS-modifying enzymes show tissue-specific expression profiles and play distinct roles in regulating the malignant behaviors of cancer cells. For instance, CHSY1 was upregulated in HCC and glioma tissues, and its upregulation was associated with poor outcomes in patients [20,21]. The CS epimerase DSE is also frequently upregulated in human gliomas, and its increased expression is typically associated with worse prognoses [22]. In contrast, DSE is frequently downregulated in HCC tissues [23]. Previous studies have indicated that CHPF is frequently upregulated in certain cancer types, and it may have cancer-promoting functions [24,25,26]. To further illustrate the functions of CS synthases during HCC progression, herein, we examined the role of CHPF, which forms the elemental structure of CS in HCC. We evaluated the correlation between clinicopathological parameters and expression of CHPF in HCC patients and explored the potential molecular mechanisms underlying its action during cancer progression. Intriguingly, we discovered that CHPF displays tumor suppressing functions in HCC cells, which is different from other types of cancer cells.

## 2. Results

### 2.1. Downregulation of CHPF in HCC

**Figure 1:**
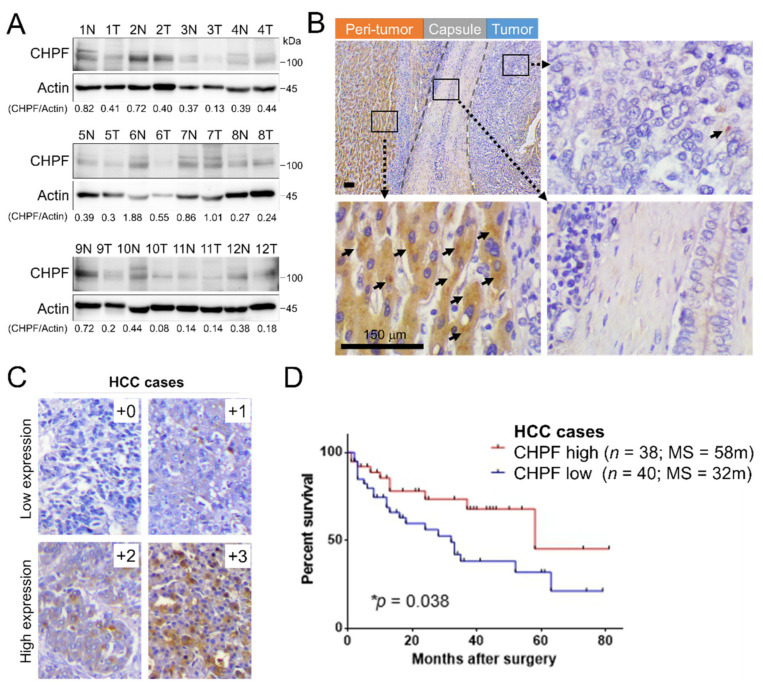
CHPF expression in human hepatocellular carcinoma (HCC). (**A**) CHPF levels and quantification in non-tumor (peri-tumor) liver (N) and tumor tissue (T) of primary HCC tissues from twelve patients. (**B**) Immunohistochemistry of CHPF in a representative HCC tissue. Dot-like precipitations of CHPF (arrow) were observed mainly in peri-tumor tissues but rarely in stromal and tumor tissues. The scale bar indicates 50 μm. (**C**) Representative images of four grades of CHPF expression in primary HCC tissue (+0, negative; +1, <20%; +2, 20–50%; +3, >50%); *n* = 78. The scale bar indicates 150 μm. (**D**) Kaplan–Meier analysis of overall survival of HCC patients. The analyses were conducted according to the immunostaining of CHPF low expression (+0 and +1) and high expression (+2 and +3); *p* = 0.038.

To explore the expression levels of CHPF in HCC tissues and non-cancerous liver tissues, we first measured CHPF protein levels in twelve primary HCC tissues using Western blotting. Each sample was divided into adjacent peri-tumor tissues and tumor tissues. The results indicated that CHPF protein levels were significantly downregulated in the tumor tissues, compared to their non-tumor counterparts (Paired *t*-test, *p* = 0.032; Figure 1A). To further examine CHPF expression, 78 HCC tissue sections procured from the Chung Shan Medical University Hospital Tissue Bank were used. Immunohistochemistry revealed that dot-like precipitates of CHPF mainly occurred in the cytoplasm of adjacent non-tumor hepatocytes but were rarely observed in the surrounding stromal cells and bile duct cells (Figure 1B). To assess the relationships between CHPF expression and clinicopathological findings in HCC patients, staining intensity was scored according to the percentage of CHPF-positive parenchymal cells per sample (0, negative; +1, <20%; +2, 20–50%; +3, >50%; Figure 1C). Our results revealed that 69% of the non-tumor liver tissues expressed high CHPF levels (+2 to +3), whereas in tumor tissues, this proportion was significantly decreased (49%; Table 1). Moreover, decreased CHPF expression was correlated with advanced tumor stages (Fisher’s exact test; *p* = 0.0202). A Kaplan–Meier survival analysis showed that survival rates of HCC patients with low CHPF expression (+0 to +1) was significantly lower than those of patients with high CHPF expression (+2 to +3; log-rank test; *p* = 0.038; Figure 1D). To confirm this observation, CHPF immunohistochemistry was performed on an independent HCC cohort (*n* = 89). The results indicated that CHPF was downregulated in HCC tissues compared to non-tumor liver specimens. Consistently, low CHPF expression in HCC was significantly correlated with low survival rates (Figure S1A,B; Table S1). Taken together, our results suggest that CHPF is frequently downregulated in HCC, and that this downregulation is associated with advanced tumor stages and poor survival chances in HCC patients.

### 2.2. CHPF Affects HCC Cell Growth In Vitro and In Vivo

**Figure 2:**
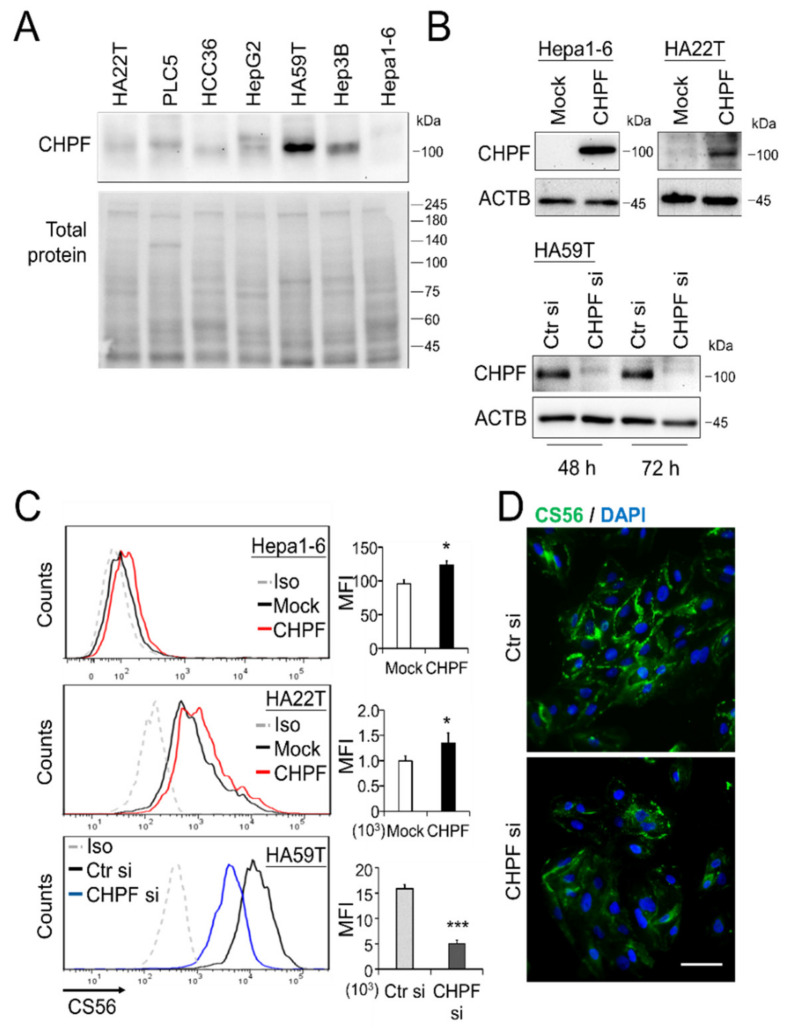
CHPF expression in HCC cells. (**A**) CHPF expression in seven HCC cell lines. Protein expression was analyzed by Western blotting, and total protein was used as an internal loading control. (**B**) Stable CHPF overexpression in Hepa1-6 and HA22T cells. Transient CHPF knockdown in HA59T cells 48 and 72 h after siRNA transfection. Protein expression was analyzed by Western blotting, and β-actin (ACTB) was used as an internal control. (**C**) Surface CS56 antibody staining (an anti-chondroitin sulfate antibody) on Hepa1-6, HA22T, and HA59T transfectants was analyzed by flow cytometry with anti-mouse IgM-FITC. Nonspecific mouse IgM was used as an isotype control (Iso). * *p* < 0.05, *** *p* < 0.001. (**D**) Fluorescence microscopy analysis of CS56 (green); DAPI (blue) indicated the position of the nucleus. The scale bar indicates 50 μm. The cells were fixed with paraformaldehyde without permeabilization.

**Figure 3:**
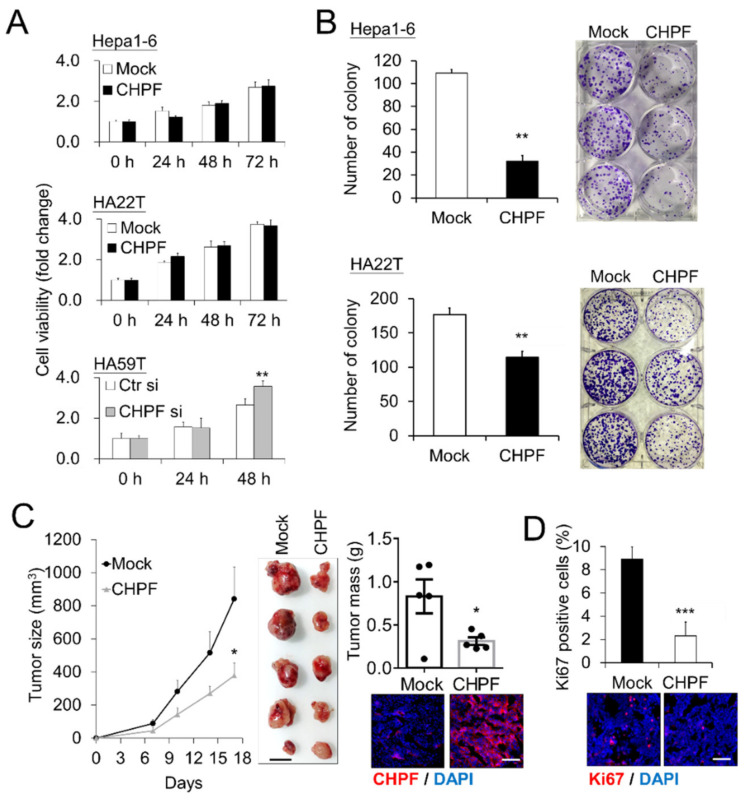
Effects of CHPF on HCC cell growth in vitro and in vivo. (**A**) CHPF modulated cell viability in vitro. Viability of Hepa1-6, HA22T, and HA59T cells was measured using CCK8 assays at indicted time points. Relative fold changes are shown. Data are presented as the means from three independent experiments ± standard deviations; ** *p* < 0.01. (**B**) CHPF overexpression suppressed colony formation in Hepa1-6 and HA22T cells. The data are presented as the means ± standard deviations; ** *p* < 0.01. Representative images are shown on the right. (**C**) Effects of CHPF on HCC tumor growth in mouse models. Hepa1-6 control transfectants and an equal number of CHPF transfectants were subcutaneously administered to the right flank (*n* = 5, each group). Size and mass of the excised tumors were recorded; shown are the means ± standard errors of the means; * *p* < 0.05. The scale bar indicates 10 mm. Immunostaining of CHPF in tumor sections (lower right; the scale bar indicates 100 μm). (**D**) CHPF suppressed cell proliferation in Hepa1-6 tumors. Proliferation of tumor cells was evaluated by immunofluorescence staining for Ki67, and representative images of tumor section are shown (bottom). The data are presented as the means from five fields of each section ± standard deviations; *** *p* < 0.001. The scale bar indicates 100 μm.

To investigate the potential effects of CHPF on malignant HCC cell phenotypes, we measured the CHPF protein levels in seven HCC cell lines and found that HA22T, HCC36, and Hepa1-6 expressed lower CHPF levels, whereas Hep3B and HA59T expressed higher CHPF levels (Figure 2A). We subsequently induced stable CHPF overexpression in HA22T and Hepa1-6 cells, and siRNA-mediated knockdown of CHPF in HA59T cells (Figure 2B). The CS was examined by anti-chondroitin sulfate antibody (CS56) staining and was quantified by flow cytometry. Although CS56 only reacts with highly sulfated CS [17,27], we found that overexpression of CHPF in Hepa1-6 and HA22T cells slightly increased the intensity of CS56 staining, which decreased in CHPF knockdown HA59T cells (Figure 2C). Immunostaining of CS56 on the cell surface also indicated that CHPF knockdown substantially decreased CS formation (Figure 2D). The CCK8 assay showed no significant effect of CHPF overexpression on Hepa1-6 and HA22T cell viability; however, CHPF knockdown slightly increased HA59T cell viability (Figure 3A). Furthermore, we observed that CHPF overexpression in Hepa1-6 and HA22T cells suppressed colony formation (Figure 3B). To investigate the effects of CHPF on tumor growth in vivo, we subcutaneously transplanted CHPF-overexpressing Hepa1-6 cells and control cells into NOD/SCID mice, and CHPF overexpression significantly decreased tumor weight and volume (Figure 3C). Immunostaining of excised tumor sections revealed a decrease in the proportion of Ki67-positive cells in CHPF-overexpressing tumor tissues, indicating that CHPF may modulate HCC cell proliferation (Figure 3D).

### 2.3. CHPF Regulates the Migration, Invasion, and Metastasis of HCC Cells

**Figure 4:**
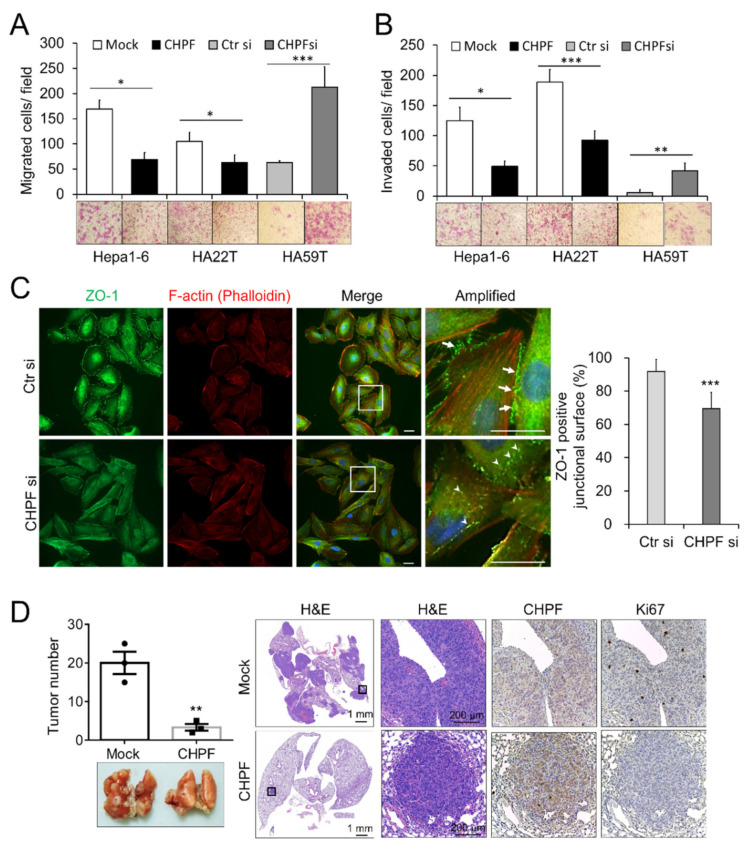
Influence of CHPF on the migration, invasion, and metastasis of HCC cells. (**A**) CHPF regulated transwell cell migration and (**B**) Matrigel invasion. CHPF overexpression significantly suppressed the migration and invasion abilities of Hepa1-6 and HA22T cells, and CHPF knockdown increased the migration and invasion abilities of HA59T cells. The data presented as the means from three independent experiments ± standard deviations. Representative images are shown at the bottom; * *p* < 0.05; ** *p* < 0.01; *** *p* < 0.001. (**C**) Immunostaining of ZO-1 (green) and F-actin (red) in HA59T cells. Amplified images are shown on the right. Note that ZO-1 is expressed on the plasma membrane of two neighboring control cells (indicated by arrows); CHPF knockdown increased the intracellular distribution of ZO-1 (indicated by arrowheads). The scale bar indicates 20 μm. The percentage of plasma membrane-associated ZO-1 positive cells was shown at right. *** *p* < 0.001. (**D**) CHPF suppressed lung metastasis. The number of tumors was decreased in the CHPF-overexpressing group. Representative images are shown at the bottom. Representative images of the hematoxylin and eosin staining and immunohistochemical staining for CHPF and Ki67 in the lung sections are shown on the right; ** *p* < 0.01.

Transwell assays were used to study the effects of CHPF on migration and invasion of HCC cells. Our results indicated that CHPF overexpression significantly reduced FBS-induced migration and invasion abilities of Hepa1-6 and HA22T cells, whereas CHPF knockdown significantly promoted the migration and invasion abilities of HA59T cells (Figure 4A,B). A decrease in the expression of the tight junction protein ZO-1 is known to be associated with the invasive behavior of HCC cells [28,29,30]; therefore, we examined whether CHPF would alter ZO-1 expression in HCC cells. Immunostaining indicated that ZO-1 was expressed on the plasma membrane of two adjoining control cells, whereas CHPF knockdown decreased ZO-1 expression on the junctional surface and increased its intracellular distribution (Figure 4C). To investigate the effects of CHPF on tumor cell metastasis, CHPF-overexpressing clones and normal controls were injected into the tail vein of NOD/SCID mice, which were killed after five weeks. In line with the in vitro results, we found that CHPF overexpression significantly reduced the number of metastatic tumors on the lung surface. Hematoxylin and eosin staining and immunohistochemistry of CHPF and Ki67 indicated decreased cell proliferation in CHPF-overexpressing tumors in the lung sections (Figure 4D).

### 2.4. CHPF Modulates TGF-β Signaling and Modifies the CS Chains of Decorin (DCN)

**Figure 5:**
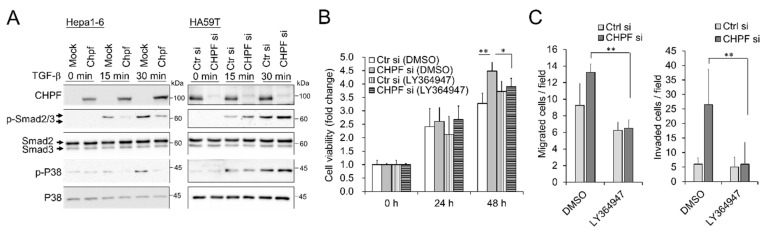
TGF-β signaling modulates CHPF-mediated malignant phenotypes. (**A**) Hepa1-6 and HA59T transfectants were starved for 3 h and then treated with (+) or without (−) TGF-β (20 ng/mL) for 15 and 30 min. Cell lysates (20 μg) were analyzed by Western blotting with various antibodies, as indicated. (**B**) Effects of LY364947, a TGF-β inhibitor, on CHPF-mediated cell viability. HA59T transfectants were treated with a solvent (0.02% DMSO) or LY364947 (0.5 μM) and then analyzed by CCK8 assay. * *p* < 0.05, ** *p* < 0.01. (**C**) Effects of LY364947 on CHPF-mediated migration (left) and invasion (right). HA59T transfectants were treated with a solvent (0.1% DMSO) or LY364947 (2.5 μM) and then analyzed by transwell migration and invasion assays. The data are shown as the means ± standard deviations. ** *p* < 0.01.

To elucidate the possible mechanisms by which CHPF regulates the malignant behavior of HCC cells, we compared differentially expressed genes in CHPF-overexpressing cells and normal controls using RNA sequencing. Gene set enrichment analysis revealed several pathways that may be involved in this process, and we observed that the TGF-β pathway can be suppressed in CHPF-overexpressing cells (Figure S2). The TGF-β pathway is crucial for regulating ZO-1 expression [31,32], and it is associated with the invasion and metastasis of HCC [33]. To examine whether CHPF regulates TGF-β signaling, HCC cells were stimulated using TGF-β (20 ng/mL), and downstream signaling was analyzed using Western blotting. Our results indicated that CHPF overexpression attenuates TGF-β-induced Smad2 and P38 activation, whereas CHPF knockdown enhances TGF-β-induced signaling (Figure 5A). Importantly, using the TGF-β type I receptor kinase inhibitor, LY364947 successfully reversed CHPF-silencing-enhanced cell viability, cell migration, and invasion (Figure 5B,C), suggesting that TGF-β signaling participates in CHPF-regulated malignancy of HCC cells. 

**Figure 6:**
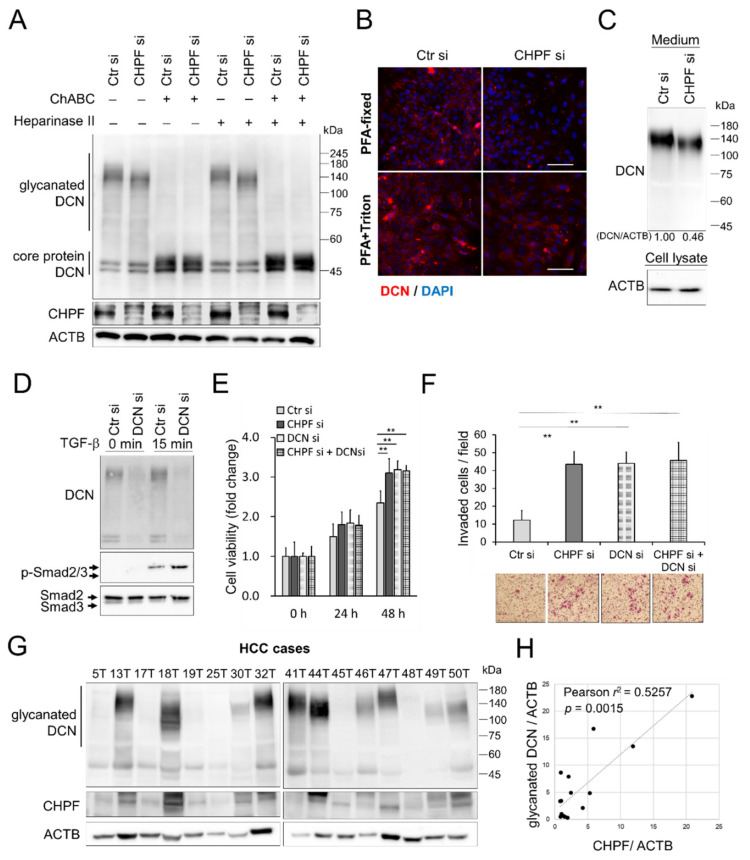
Decorin is modified by CHPF and is involved in malignant phenotypes in HCC cells. (**A**) CHPF modified the CS chains on decorin (DCN). The molecular weight of glycanated decorin was decreased after CHPF knockdown in HA59T cells. Glycanated decorin was mostly shifted to the size of core protein after 2 h of digestion with chondroitinase ABC (ChABC). These results showed no difference with regard to heparinase II treatments. Blotting of ACTB was used as an internal control. (**B**) Immunostaining of DCN in controls and CHPF-silenced cells. Cells with paraformaldehyde (PFA) fixation are shown at the top. Images of PFA fixation, followed by triton X100 permeabilization, are shown at the bottom. The scale bar indicates 50 μm. (**C**) Secreted DCN in HA59T culture medium (1 × 10^6^ of cells in 1 mL serum free medium for 24 h). Blotting of ACTB from attached cell lysate was used as a loading control. (**D**) Silence of DCN enhanced TGF-β signaling. Effects of DCN silencing on CHPF-mediated cell viability (**E**) and cell invasion (**F**). The HA59T cells were transfected with non-targeting control siRNAs (Ctr si), CHPF si, and/or DCN specific siRNAs (DCN si). Cells were subjected to CCK8 assay or transwell invasion assay. Data are expressed as the mean ± SD from three independent experiments. ** *p* < 0.01. The representative images are shown on the right. (**G**) Western blots of DCN and CHPF in 16 primary HCC tissues. ACTB was used as a loading control. (**H**) Correlations between CHPF protein expression and glycanated DCN in HCC tissues; Pearson *r*^2^ = 0.5257; *p* = 0.0015.

CS synthases-remodeled CS proteoglycans (CSPGs) are crucial for modulating signal transduction and cell–cell communication [21,23,34], and CSPG DCN is known to be a key regulator of TGF-β signaling [35,36,37]. Thus, we examined the influence of CHPF on DCN in HCC cells. Western blotting revealed that CHPF knockdown decreased the molecular weight of glycosaminoglycan-containing (glycanated) DCN in HA59T cells (Figure 6A), whereas CHPF overexpression increased the levels of glycanated DCN in HA22T cells (Figure S3A). Moreover, glycanated DCN diminished to the size of DCN core protein only when the GAG chains were removed by chondroitinase ABC, indicating that CHPF knockdown shortens the CS chains on DCN. To examine the effects of CHPF on DCN distribution, we performed immunostaining of DCN on HCC cells with or without cellular permeabilization, which showed that CHPF knockdown substantially decreased the distribution of extracellular DCN surrounding cells, whereas intracellular DCN was not significantly affected (Figure 6B). In contrast, CHPF overexpression increased extracellular DCN distribution on HA22T cells (Figure S3B). Consistently, the protein levels of secreted DCN were decreased in CHPF knockdown medium (Figure 6C); whereas they increased in CHPF overexpressed conditional medium (Figure S3C). We suggested that CHPF-modified DCN may enhance its stability surrounding HCC cells. To further examine the contribution of DCN in TGF-β signaling and CHPF-regulated phenotypical changes, we silenced DCN expression by siRNA. The silencing of DCN enhanced TGF-β signaling in HCC cells (Figure 6D). Additionally, silencing of DCN increased cell viability and cell invasion in control cells, but did not have further influences on CHPF knockdown cells (Figure 6E,F). We subsequently evaluated the correlation between CHPF expression and DCN in HCC tissues. Western blotting indicated that CHPF expression was positively correlated with the expression of glycanated DCN (Figure 6G,H), suggesting that CHPF-modified DCN may increase glycanated DCN accumulation in HCC tissues.

## 3. Discussion

Several recent studies have reported that CHPF is associated with cancer progression. For instance, CHPF knockdown may inhibit glioma cell growth [24], and CHPF upregulation promotes the proliferation of, and anti-apoptosis in, lung adenocarcinoma cells [25]. Moreover, CHPF can promote the tumorigenicity of malignant melanoma by regulating CDK1 [26]. Although such a tumor-promoting role of CHPF in certain types of cancer has been proposed, the direct effects of CHPF-modified CS have not yet been examined. CS covalently binds to PGs and helps them perform their normal biological functions. We reason that the expression of PGs in different cancer types may govern the influence of CHPF on cancer cells. For instance, versican was observed in various types of malignancies, including breast cancer, prostate cancer, and HCC, where it is associated with high rates of cancer relapse and poor outcomes [38,39,40,41]. In contrast, DCN and protein proteoglycan 4 demonstrate protective functions in HCC cases [42,43]. Our results demonstrate that DCN is one of the CSPGs that are modified by CHPF, and the shortened CS on DCN may alter its extracellular distribution around HCC cells.

A recent study on CHPF in HCC reported high CHPF expression in 45% (35/77) of HCC cases and low CHPF expression in all para-carcinoma tissues, which was observed using immunohistochemistry [44]. In the present study, we evaluated the expression of CHPF using Western blotting and immunohistochemistry in HCC cases treated at a local hospital and using a commercial human liver cancer tissue microarray. Our results consistently indicated that CHPF is frequently downregulated in tumor tissues, compared to the case for the adjacent non-tumor liver tissues. In addition, CHPF downregulation is associated with reduced survival spans of HCC patients. Moreover, our results demonstrate that restoring CHPF expression can decrease HCC cell growth, migration, and invasion in vitro and in vivo, and CHPF silencing in HA59T cells enhanced malignancy. Taken together, this confirms the tumor-suppressive effects of CHPF in HCC cells. We cannot rule out that the discrepancy within these studies may result from the different cell lines and HCC cohorts we used. We also supposed that the different results of immunohistochemistry may be due to the use of different antibodies for immunohistochemistry. The monoclonal antibody against CHPF used in our study produced clear cytoplasm and para-nuclear staining patterns in parenchymal cells and very little background signal in connective tissues and stromal cells (Figure 1B).

Previous studies indicated that CS synthases achieved CS polymerization via multiple combinations of CHSY1, CHSY3, and CHPF [18,19]. A different study using CHPF-knockout chondrocyte culture systems found that CHPF mainly participated in the extension of CS, whereas CHSY1 participated in both extension and initiation of CS [45]. Our results showed that CHPF knockdown decreased the size of CS on DCN but did not completely diminish the glycanated DCN, suggesting that other CS synthases also participate in CS formation on DCN, and one major function of CHPF appears to be CS extension. This result is in line with those of previous studies suggesting that each CS synthase may play a unique role in the biosynthesis of CS.

CSPG DCN is commonly expressed throughout collagenous tissues and is crucial for the adequate functioning of collagen fibers. DCN is also known as a tumor suppressor. p53- and DCN-double-knockout mice show a significantly faster rate of lymphoma development than signal p53-knockout mice [46]. Recombinant DCN, in combination with celecoxib, potentially suppresses epithelial-mesenchymal transition in colorectal cancer [47]. Reduced expression or total absence of DCN has been observed in various forms of human cancers, including HCC [48,49,50]. A previous report indicated that DCN gene delivery reduced liver tumor formation in a mouse model [43]. Tumor-suppressive effects of DCN are attributed to the neutralizing nature of different growth factors that have been shown to bind and inhibit the activity of all three mammalian TGF isoforms TGF-β1, -β2, and -β3, even when bound to collagen [51]. However, the influence of CS on the bioactivity of DCN has not been thoroughly investigated. Our results indicate that CHPF knockdown shortens the size of CS on DCN, which may attenuate the inhibition of TGF-β signaling. A previous study also suggested that glycosaminoglycan chains may impede interactions of DCN core proteins with TGF-β [36]. In addition, a recent report suggested that blocking DCN secretion results in increased activation of TGF-β [52]. Our immunostaining results of DCN in HCC cells suggested that CS on DCN may facilitate its extracellular accumulation. These findings confirm the assumption that the status of CS chains on DCN may modulate their tumor-suppressive functions in the TME. 

TGF-β has been reported to stimulate CHPF expression in nucleus pulposus cells by activating the Smad3, RhoA/ROCK1, and MAPK signaling pathways [53]. Our study demonstrates that CHPF elongates the CS chain on DCN and may subsequently attenuate TGF-β signaling. We thus conclude that the CS chain on DCN participates in a negative feedback loop with TGF-β signaling and CHPF expression. This may be an important mechanism for maintaining the homeostasis of TGF-β signaling in animal tissues. 

## 4. Materials and Methods

### 4.1. Reagents and Antibodies

Recombinant TGF-β1 protein was purchased from PeproTech. Full length CHPF cDNA clone was purchased from OriGene. Mouse monoclonal antibody against CHPF (sc-376183) was purchased from Santa Cruz. Antibodies against p-Smad2/3 (#8828), Smad2/3 (#8658), p-AKT (#4060), AKT (#4691), p-p38 (#9211), p38 (#9212), and ZO-1 (#8193) were purchased from Cell Signaling Technology, Inc. Goat polyclonal anti-decorin (R&D Systems, catalog #AF143) was used to examine expression of Decorin. Antibodies against actin were purchased from GeneTex, Inc. (Irvine, CA, USA) CS56 antibody (C8035) was purchased from Sigma-Aldrich. The dilutions of antibodies were 1 in 2000 for Western blots, and 1 in 200 for immunostaining and flow cytometry. 

TGF-β inhibitor, LY364947 (Cayman Chemical, Ann Arbor, MI, USA) was dissolved in DMSO (1.0 mg/mL) and applied to inhibit TGF-β receptor. Chondroitinase ABC and CCK8 reagent were purchased from Sigma-Aldrich. Heparinase II was purchased from R&D Systems.

### 4.2. Human Tissue Samples

Post-surgery frozen and paraffin-embedded HCC tissues for Western blots and immunohistochemistry were obtained from the Chung Shan Medical University Hospital (Taichung, Taiwan). This study was approved by the Ethical Committees of Chung Shan Medical University Hospital, and all patients gave informed consent to have their tissues before collection (CSMUH No: CS218075). Commercial human paired HCC tissue microarray with survival data (HLiv-HCC180Su) was purchased from Shanghai Outdo Biotech (Shanghai, China) and Pantomics, Inc (Fairfield, CA, USA).

### 4.3. Immunohistochemistry

Paraffin-embedded HCC tissues were incubated with anti-CHPF antibody (1:200) in 5% bovine serum albumin/PBS and 0.1% Triton X-100 (Sigma) for 16 h at 4 °C. UltraVision Quanto Detection System (Thermo Fisher Scientific Inc. Waltham, MA, USA) was used to amplify the signal. The specific immunostaining was visualized with 3,3-diaminobenzidine and counterstained with hematoxylin (Sigma, St. Louis, MO, USA). Images were scanned by TissueFAX Plus Cytometer. The distribution and positive intensity of CHPF were graded by two scorers blinded to the clinical parameters.

### 4.4. Cell Culture

HCC cell lines, HA22T, PLC5, HepG2, HA59T, Heb3B, and Hepa1-6, were purchased from Bioresource Collection and Research Center in the year 2014 (Hsinchu, Taiwan). HCC36 cells were ungrudgingly furnished by Prof. Lei Wan (China Medical University). Cells above were cultured in DMEM containing 4.0 mM L-glutamine and 10% FBS in 5% CO_2_ at 37 °C.

### 4.5. Transfection and RNA Interference 

For overexpression experiments, Hepa1-6 cells and HA22T cells were transfected with CHPF plasmids using Lipofectamine-3000 (Invitrogen, Carlsbad, CA, USA), and empty pCMV6 plasmid was used as mock transfectant. Cells were selected with 400 μg/mL of G418 for 14 days. For siRNA knockdown experiments, ON-TARGETplus SMARTpool siRNA against CHPF, DCN, and control siRNA were purchased from Dharmacon. RNAiMAX (Thermo Fisher Scientific Inc., Waltham, MA, USA) was used for transfection and 20 nM of siRNA was used in all knockdown experiments.

### 4.6. Cell Invasion and Migration Assay

Transwell inserts for 24-well plate (Corning, Glendale, AZ, USA) with uncoated porous filters (pore size 8 mm) were used to evaluate cell migration, and Matrigel (BD Biosciences, San Jose, CA, USA) coated porous filters were used to examine cell invasion. Hepa1-6 (2 × 10^4^ cells), HA22T (2 × 10^4^ cells), and HA59T (5 × 10^4^ cells) in 0.1 mL serum-free DMEM were seeded into inserts, and 0.6 mL DMEM containing 10% FBS was added in lower part of the well. Hepa1-6 cells, HA22T cells, and HA59T cells were incubated for 24 h. Cells on the upper side of the membrane were wiped. Cells moving to the other side of the filters were stained by crystal violet and counted manually. Values were shown as the average number of cells per microscopic field over three fields of each filter. Independent experiments were repeated three times. 

### 4.7. Cell Proliferation and Colony Formation

Cells (2 × 10^3^) were seeded into 96-well plates with culture medium. Living cells were analyzed by CCK8 at 0, 24, 48, and 72 h. For the anchorage-dependent colony formation assay, 500 cells were seeded in 6-well plates and incubated for 20 days. Colonies were fixed in methanol and stained with 0.1% crystal violet (Sigma, St. Louis, MO, USA) for counting. The number of anchorage colonies was counted manually.

### 4.8. Immunofluorescence

Cells were cultured in cover slips and fixed with 4% paraformaldehyde for 15 min at room temperature. The samples were blocked in 5% BSA-PBS for 1 h with or without 0.1% of Triton X100 (Sigma) for cell membrane permeabilization. Cells on cover slips and frozen sections of Hepa1-6 tumor tissue were stained with antibody against CS (CS56, #C8035; 1:200; Sigma), Ki67 (#ab1667; 1:100; Abcam), and CHPF. Cell Navigator^TM^ F-actin Labeling Kit (1:1000, AAT Bioquest Inc., Sunnyvale, CA, USA) was used to visualize F-actin. DAPI was used for nuclear staining. Images were captured by ZEISS Axio Imager A2 microscope. The plasma membrane-associated ZO-1 positive cells were analyzed by microscope images (400× magnification), and 450–500 cells from 10 randomly selected fields were evaluated in each sample.

### 4.9. Western Blotting

Total protein lysates (20–40 μg) were separated by 8% SDS-PAGE and transferred to PVDF membrane. Total protein was measured by stain-free technology (Bio-Rad, Hercules, CA, USA). To analyze TGF-β-triggered signaling, cells were serum-starved for 3 h and then stimulated with 20 ng/mL of TGF-β for 15 min or 30 min. For the digestion of GAGs, protein lysates (40 μg) were pretreated with 0.5 units/mL protease-free chondroitinase ABC (Sigma) or heparinase II (R&D Systems, Minneapolis, MN, USA) for 2 h at 37 °C. Intensity of signals on Western blotting was quantified by ImageJ software (version: 1.53 g, U. S. National Institutes of Health, Bethesda, MD, USA) (Wayne Rasband).

### 4.10. Metastasis and Tumor Growth Mice Models 

Male NOD/SCID mice, 5 weeks of age, were purchased from National Laboratory Animal Center (Tainan, Taiwan). For tumor growth analysis, 3 × 10^6^ of Hepa1-6 mock transfectants and equal number of CHPF transfectants were subcutaneously and respectively inoculated into left and right flank (*n* = 5, both group). Tumor volumes were monitored for 15 days. Excised tumors were weighed and analyzed. For the tumor metastasis model, Hepa1-6 stable transfectants were injected into tail veins of mice (1 × 10^6^ cells/mouse, *n* = 3 for each group). Mice were sacrificed 5 weeks after inoculation. The lungs were excised and surface nodules were counted. Excised tissues were paraffin-embedded for H&E stain and IHC staining.

All animal experiments in this study were reviewed and approved by the Institutional Animal Care and Use Committee (IACUC) of Chung Shan Medical University Experimental Animal Center.

### 4.11. Statistical Analysis

All data analyses were performed using GraphPad Prism 6. Student t-test was used for statistical analyses. Paired t-test was used for the analyses of paired HCC tissue and paired Hepa1-6 tumors. Two-sided Fisher exact test was used for comparisons between CHPF expression and clinicopathologic features. Kaplan–Meier analysis and the log-rank test were used to estimate overall survival. *p* < 0.05 was considered statistically significant.

## 5. Conclusions

In conclusion, our results suggest that CHPF can modify the CS chain on DCN and regulate TGF-β signaling, thereby modulating the malignancy of HCC cells. This not only implies a pathophysiologic role of CHPF in HCC cells, but also suggests significance of abnormal CS during HCC tumor progression. Understanding the biological functions and effects of CS on DCN as a tumor suppressor and on other PGs that are modified by CS synthases may offer a novel strategy for developing HCC therapeutic agents. These include recombinant CSPG, siRNAs, CSPG synthesis inhibitors, and CS degradation enzymes.

## Figures and Tables

**Table 1:** Correlation of CHPF expression with clinicopathological features of hepatocellular carcinoma tissue.

Factor	Feature	CHPF Expression		*p* Value (Two-Sided Fisher’s Exact Test)
		Low	High	
Tissue types	Non-tumor	15	34	0.0275 *
	Tumor	40	38	
Sex	Male	31	29	1.00
	Female	9	9	
Age	<55 years	10	7	0.5869
	≥55 years	30	31	
Tumor stage	T1 + T2	20	29	0.0202 *
	T3 + T3	20	9	
Metastasis	No	1	0	1.00
	Yes	39	38	

* *p* < 0.05 was considered as statistically significant.

## Data Availability

The data presented in this study are available on request from the corresponding author. The data are not publicly available due to ethical issues.

## Supplementary Materials

The following are available online at https://www.mdpi.com/2072-6694/13/6/1261/s1, Figure S1: (A) Immunohistochemistry of CHPF in a paraffin-embedded human HCC tissue microarray; (B) Kaplan–Meier analysis of overall survival for patients with HCC, Figure S2: Gene Set Enrichment Analysis (GSEA) of deferentially expressed genes in control cells and CHPF overexpressed cells using hallmark gene sets, Figure S3: CHPF modified CS chains on decorin(DCN) in HA22T cells, Table S1: Correlation of CHPF expression with clinicopathological features of HCC tissue array.

## Abbreviations

CHPF	Chondroitin polymerizing factor
CS	chondroitin sulfate
DCN	decorin
GAGs	Glycosaminoglycans
GalNAc	*N*-acetylgalactosamine
HCC	hepatocellular carcinoma
PGs	proteoglycans
TME	tumor microenvironment
