# Supplementary material for: CHPF Regulates the Aggressive Phenotypes of Hepatocellular Carcinoma Cells via the Modulation of the Decorin and TGF-β Pathways
Source: Cancers (Basel). 2021 Mar 12;13(6):1261. doi: 10.3390/cancers13061261 (PMC8002199; doi:10.3390/cancers13061261)
Supplement: Supplementary file 1 [file cancers-13-01261-s001.pdf]

Supplementary Materials

CHPF Regulates the Aggressive Phenotypes of Hepatocellular Carcinoma Cells via the Modulation of the Decorin and TGF-β Pathways

Chiung-Hui Liu, Bo-Rui Wu, Ying-Jui Ho, Yin-Hung Chu, Wei-Cheng Hsu, To-Jung Tseng, Ju-Pi Li and Wen-Chieh Liao

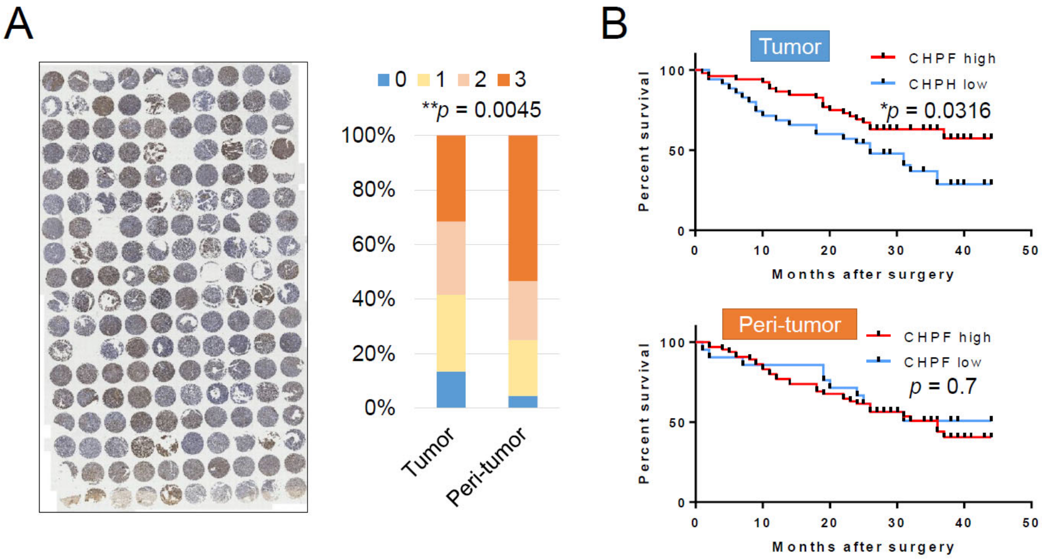

Figure S1. (**A**) Immunohistochemistry of CHPF in a paraffin-embedded human HCC tissue microarray. The results were scored based on the percentage of positive cells in the tumor and peri-tumor tissue (0, <5%; 1, 5 to 20%; 2, 20% to 50%; 3, >50%). $p = 0.0045$. (**B**) Kaplan-Meier analysis of overall survival for patients with HCC. The analyses were conducted according to the immunohistochemistry of CHPF. Grade 0 and 1 for low expression; Grade 2 and 3 for high expression. Log-rank test. $p = 0.0316$; $p = 0.7$.

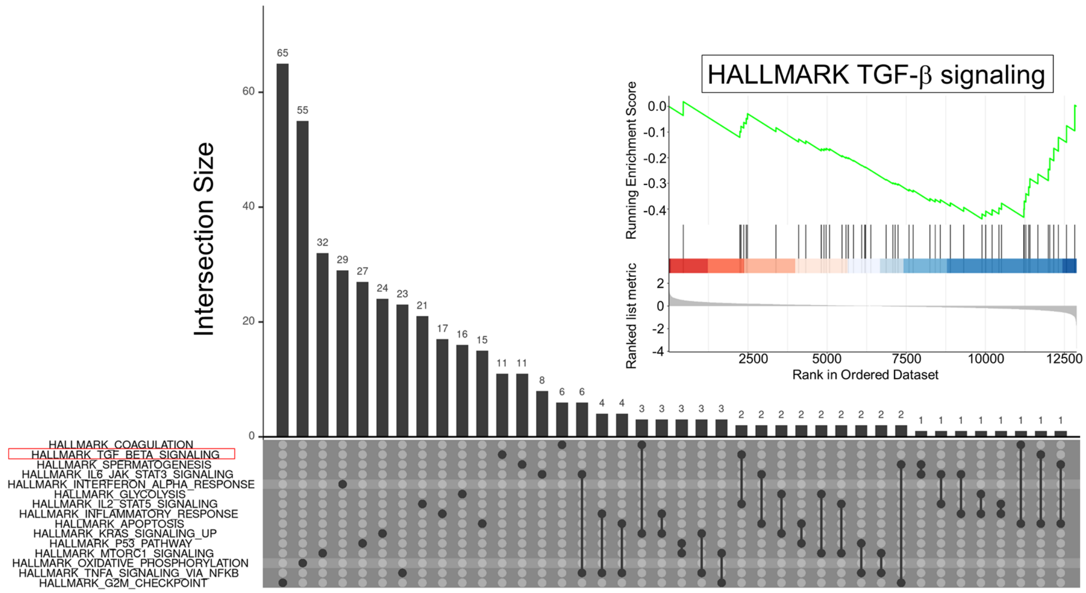

Figure S2. Gene Set Enrichment Analysis (GSEA) of deferentially expressed genes in control cells and CHPF overexpressed cells using hallmark gene sets. Intersection size plot of significantly different pathways are ranking at left. Downregulated genes plot of TGF-b signaling was shown at upper right.

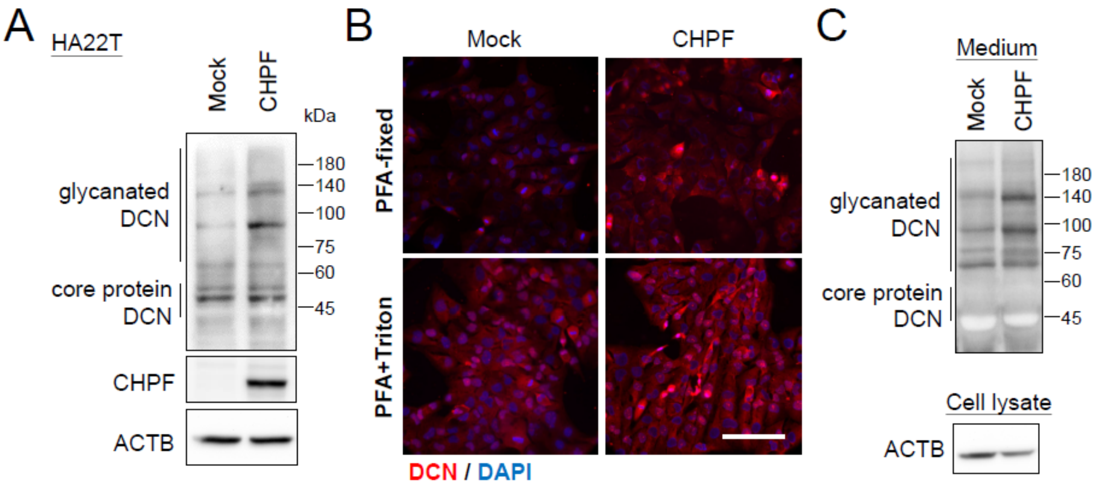

Figure S3. CHPF modified CS chains on decorin(DCN) in HA22T cells. (**A**) Glycanateddecorinwas increased in CHPF overexpressed cells. β-actin (ACTB) was used as an internal control. (**B**) Immunostaining of DCN in control vector (mock) and CHPF overexpressed cells. Cells with paraformaldehyde (PFA) fixation were shown at top. Images of PFA-fixed followed by triton X100 permeablizationwere shown at bottom. Scale bar 50 μm. (**C**) Secreted DCN in HA22T culture medium (1×10^6 of cells in 1 mL serum free medium for 24 h). Blotting of ACTB from attached cell lysate was used as a loading control.

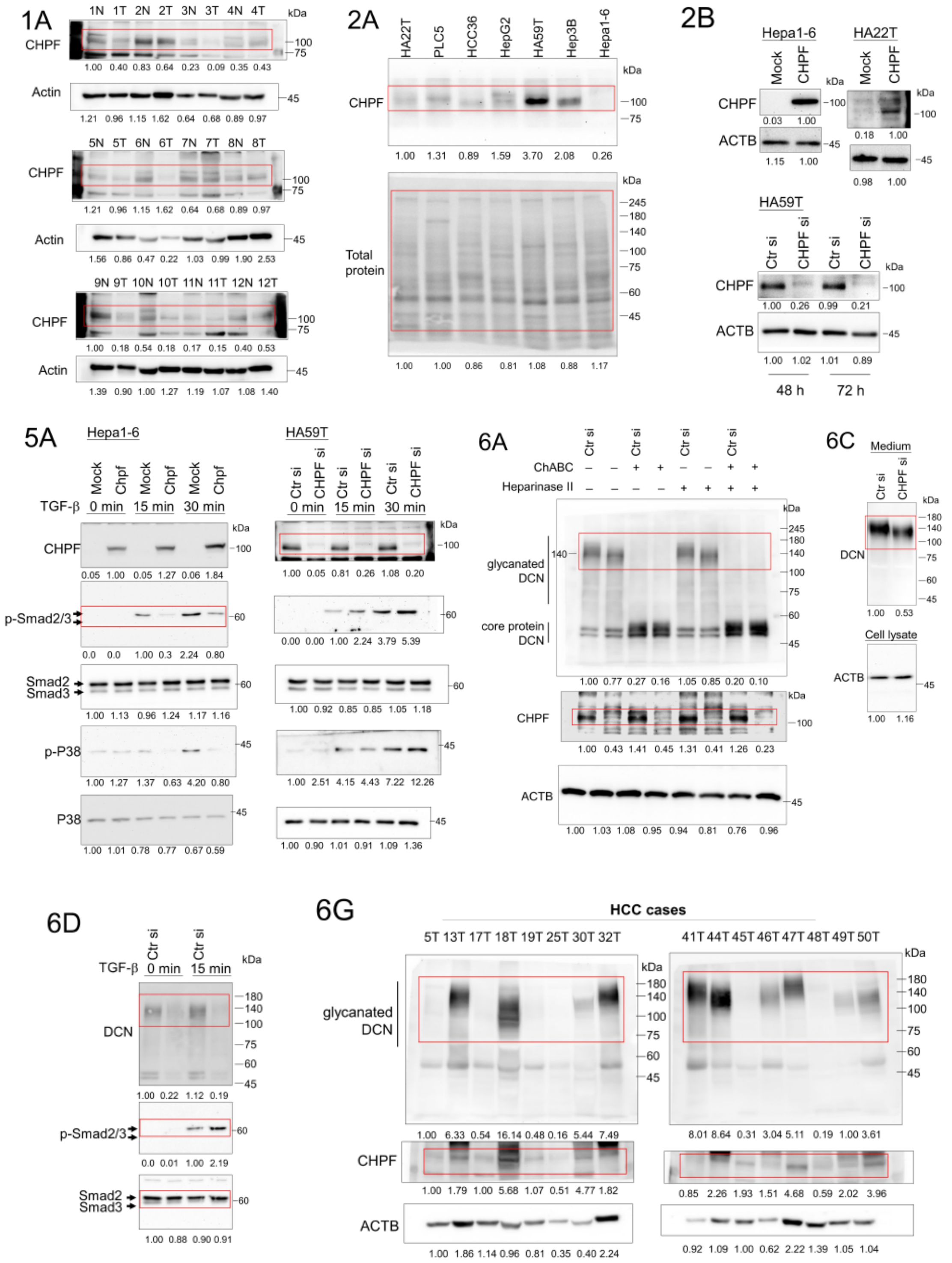

Detail information about Figures 1,2,5,6.

Table S1. Correlation of CHPF expression with clinicopathological features of HCC tissue array.

Factor	Feature	CHPF Expression		*p* Value (Two-Sided Fisher's Exact Test)
		Low	High	
Tissue types	Non-tumor	22	66	0.0045*
	Tumor	37	52	
Sex	Male	29	45	1.00
	Female	6	9	
Age	< 55 years	20	24	0.2820
	≥ 55 years	15	30	
Tumor stage	T1 + T2	17	34	0.2433
	T3 + T4	15	16	
Metastasis	No	35	54	1.00
	Yes	0	0	

*$p < 0.05$ was considered as statistically significant.
